# Skin biopsies in acute myeloid leukemia patients undergoing intensive chemotherapy are safe and effect patient management

**DOI:** 10.1038/s41598-021-91576-7

**Published:** 2021-06-07

**Authors:** Tamar Berger, Shany Sherman, Lucille Hayman, Ofir Wolach, Adi Shacham-Abulafia, Pia Raanani, Oren Pasvolsky

**Affiliations:** 1 Institute of Hematology, Davidoff Cancer Center, Rabin Medical Center – Beilinson Hospital, 39 Zeev Jabotinsky St., 4941492 Petach Tikva, Israel; 2 Sackler Faculty of Medicine, Tel Aviv University, Tel Aviv, Israel; 3 Division of Dermatology, Rabin Medical Center – Beilinson Hospital, Petach Tikva, Israel; 4 Department of Pathology, Rabin Medical Center – Beilinson Hospital, Petach Tikva, Israel

**Keywords:** Haematological diseases, Haematological cancer, Pathology

## Abstract

There is paucity of data regarding the diagnostic yield and safety of skin biopsies in patients with acute myeloid leukemia (AML), though skin eruptions are common in these patients. We evaluated 216 patients treated in our hemato-oncology unit at a tertiary medical center between 2007 and 2018 and identified 35 patients who underwent 37 skin biopsies. The majority of biopsies were performed during induction treatment for AML (n = 26, 70%), whereas the remainder of biopsies were done prior to induction initiation (n = 8, 22%) or during consolidation chemotherapy (n = 3, 8%). Pathology findings were inconclusive in 13 cases (35%), while diagnostic biopsies were positive for drug eruptions (24%), leukemia cutis (16%), infections (11%), reactive processes (8%) and Sweet syndrome (5.5%). In almost half of cases (16/37) tissue cultures were performed. Of those, only a quarter (4/16) were positive. Histopathology and tissue culture results altered immediate patient care in 3 cases (8%), yet information obtained from biopsies had potential to affect long term patient care in 8 additional cases (21.6%). Although most skin biopsies were performed while patients had severe thrombocytopenia and neutropenia, only one patient had a complication due to the biopsy (fever and local bleeding). With the limitation of a retrospective analysis, our study suggests that skin biopsies in patients treated for AML are relatively safe. Although biopsy results infrequently alter immediate patient management, long term effect on patient care expand the potential diagnostic yield of skin biopsies.

## Introduction

Acute myeloid leukemia (AML) is an aggressive hematological malignancy usually presenting with an abnormal leukocyte count and thrombocytopenia, with an inherent risk of both infection and bleeding. Fit patients with AML are treated in-hospital with intensive induction chemotherapy traditionally containing cytarabine and an anthracycline. Post-remission consolidation therapy includes either an allogeneic hematopoietic stem cell transplant or additional courses of high dose cytarabine-containing chemotherapy^1,2^. Hospitalization for induction and consolidation treatments can be lengthy, mainly owing to a prolonged chemotherapy-induced period of aplasia, with subsequent potential infectious and bleeding complications.

Skin eruptions in patients with AML, both at presentation and during treatment, are commonly encountered in clinical practice. They may be drug-related or reactive rashes or due to infections, leukemia cutis, Sweet syndrome, or neutrophilic eccrine hidradenitis^3–10^. In some cases, skin biopsies are performed for histopathologic study and tissue culture in order to correctly diagnose the rash and provide tailored treatment.

Although skin biopsy is only a minor invasive procedure, there is still a risk of complications, such as infection and bleeding^11^. The risk is potentially higher in immunosuppressed, thrombocytopenic patients receiving intensive chemotherapy due to AML. Data on the prevalence and characteristics of skin eruptions in patients with AML are scarce, and even less is known about the yield and safety of skin biopsies in this setting.

The aim of this study was to evaluate the diagnostic yield and safety of skin biopsies obtained from adult patients with AML during hospitalization for intensive chemotherapy.

## Methods

The electronic database of the hemato-oncology unit of a tertiary university-affiliated medical center was retrospectively reviewed for adult patients with AML. All patients had undergone a dermatological clinical assessment and were referred for a skin biopsy for the evaluation of a skin rash during hospitalization for induction or consolidation treatments between January 1, 2007 and January 9, 2018. Skin biopsies were analyzed by a skilled dermatopathologist. Patients with AML who were not treated with intensive induction protocols were excluded. The following data were collected: demographic details, AML characteristics, chemotherapy regimens, clinical description of the skin eruption and associated symptoms (i.e., fever, pruritus), and laboratory tests including complete blood count, chemistry, and coagulation parameters at the time of biopsy. The rash was graded for severity according to the Common Terminology Criteria for Adverse Events (CTCAE), version 5^12^. Biopsy samples were evaluated by histopathology and tissue culture. We recorded whether the results had an impact on patient care, i.e.: (1) a change in antimicrobial coverage to treat a new pathogen recovered from the skin biopsy culture, (2) a diagnosis of leukemia cutis in a patient with no other evidence of leukemia, (3) initiation of steroid therapy in response to a biopsy results suggestive of an inflammatory or hypersensitivity reaction such as Sweet syndrome or drug reaction, (4) a culprit drug discontinuation in response to a diagnosis of drug reaction in skin biopsy. Complications due to skin biopsy were also documented.

### Statistical analysis

Categorical variables are presented as number and percentage, and continuous variables as median and range. Data were analyzed by standard statistical tests. Statistical analyses were performed by using Excel software.

### Ethics approval

The study was approved by the Institutional Review Board (IRB) of Rabin Medical Center (RMC). Informed consent was waived by the RMC IRB ethics committee due to the retrospective design of the study. IRB protocol approval number 0622-18-RMC. All research was performed in accordance with relevant guidelines and regulations.

## Results

Of the 216 consecutive patients diagnosed with AML at our institute during the study period, 35 developed a rash during treatment and underwent skin biopsy. All patients were clinically evaluated by a dermatologist who indicated and performed the biopsy. Patient demographics and rash characteristics are presented in Table 1.

**Table 1:** Characteristics of 35 patients undergoing 37 skin biopsies during hospitalization for intensive chemotherapy for AML.

Characteristics	Value (based on 37 biopsies)
Male gender, n (%)	18 (49)
Age (years), median (range)	59 (20–79)
**Diagnosis, n (%)**	
AML, other than APL	35 (95)
APL	2 (5)
**Biopsy timing, n (%)**	
**During induction**	
At presentation, before chemotherapy	8 (22)
During or post-induction treatment	26 (70)
During consolidation	3 (8)
Time from chemotherapy (days), median (range)*	10 (2–51)
Regimen prior to skin eruption, n (%)*	
Induction with daunorubicin + ARA-C (“3 + 7”)	23 (79)
ARA-C as consolidation	3 (10)
Salvage protocol (high dose ARA-C + mitoxantrone)	1 (4)
ATRA + idarubicin	2 (7)
**Grade of rash, n (%)**^**†**^	
1	18 (53)
2	9 (26)
3	5 (15)
4	2 (6)
**Rash morphology**	
Maculopapular	16 (43)
Nodular	4 (11)
Vesicular / pustular	4 (11)
Ecthyma	3 (8)
Purpura	1 (3)
Striae	2 (5)
Undetermined	7 (19)
**Rash distribution, n (% of total areas involved)**^**‡**^	
Head and neck	5 (11)
Trunk	15 (33)
Limbs	23 (51)
Missing data	2 (4)
**Laboratory data at time of biopsy**	
Neutrophil count (per microL), median (range)	200 (0–9700)
Neutropenia (neutrophils < 500 cells/microL), n (%)	23 (62)
Platelet count (per microL), median (range)	127,000 (6000–398,000)
PT (seconds), median (range)	15 (11–21)
PTT (seconds), median (range)	31 (17–47)
Fever at time of rash, n (%)	16 (43)
Bacteremia at time of biopsy, n (%)	4 (11)

*For the 29 skin eruptions appearing after chemotherapy commencement.

^†^Data available for 34/37 skin biopsies. Grade according to CTCAE, version 5^12^.

^‡^If rash involved more than one body part, it was counted more than once. Value represents percent of total areas involved in all patients (n = 45).

*AML* acute myeloid leukemia, *APL* acute promyelocytic leukemia, *3* + *7* daunorubicin and cytarabine, *ARA-C* cytosine arabinoside, *ATRA* all trans retinoic acid, *CTCAE* Common Terminology Criteria for Adverse Events, *PT* prothrombin time, *PTT* partial thromboplastin time.

A total of 37 biopsies were performed: 22% at presentation, prior to chemotherapy administration and 78% following the onset of chemotherapy, either after the standard daunorubicin and cytarabine (3 + 7) induction regimen (70%) or after post-induction (consolidation or salvage) treatments (8%).

Most rashes were grade 1 (53%) or 2 (26%) in severity; only 21% were grade 3 (15%) or grade 4 (6%). The most common body areas involved were the limbs (51%) and trunk (33%).

At the time of skin biopsy, 43% of patients had associated fever and 11% had documented bacteremia. Most patients (62%) had severe (grade 4) neutropenia (< 500 cells/microL), and the median neutrophil count was 200 cells/microL. In addition, 38% and 19% of patients had grade 3 and 4 thrombocytopenia (platelet count < 50,000 and < 25,000/microL), respectively. Two-thirds of patients had prolongation of prothrombin time.

The histologic biopsy findings were nondiagnostic in 35% of cases. Informative biopsies revealed drug reactions (24%), leukemia cutis (16%), infectious etiology (11%), Sweet syndrome (5%), and reactive findings (8%). In 2 cases, both with drug reactions, the biopsy results led to an immediate change in management: in one, the culprit drug was stopped, and in the other, systemic corticosteroid treatment was administered accompanied by withdrawal of empirical acyclovir.

In 16 skin biopsies (43%), a bacterial and/or fungal culture was performed. Cultures were positive in 4 cases (25%); however, only in one patient, did the positive culture (an isolate of *Acinetobacter baumannii*) result in an immediate change of the antimicrobial coverage. In the other 3 cases, the skin biopsies were positive for *Pseudomonas luteola*, which was already covered by empirical antibiotic treatment, *Enterococcus spp*. which was also isolated from the patient’s bloodstream, and *Syncephalastrum spp.* which was considered a contaminant.

Thus, overall, of the 37 skin biopsies performed, 3 (8%) led to a direct change in management. In all the patients for whom skin biopsy results altered immediate management, the biopsy was performed during or after AML treatment (as oppose to prior to initiation of AML treatment). Yet, when considering the potential to affect long-term management, biopsy yielded valuable information in an additional 8 cases (21.6%)—six with leukemia cutis and two with non-contaminant infectious etiologies.

**Figure 1:**
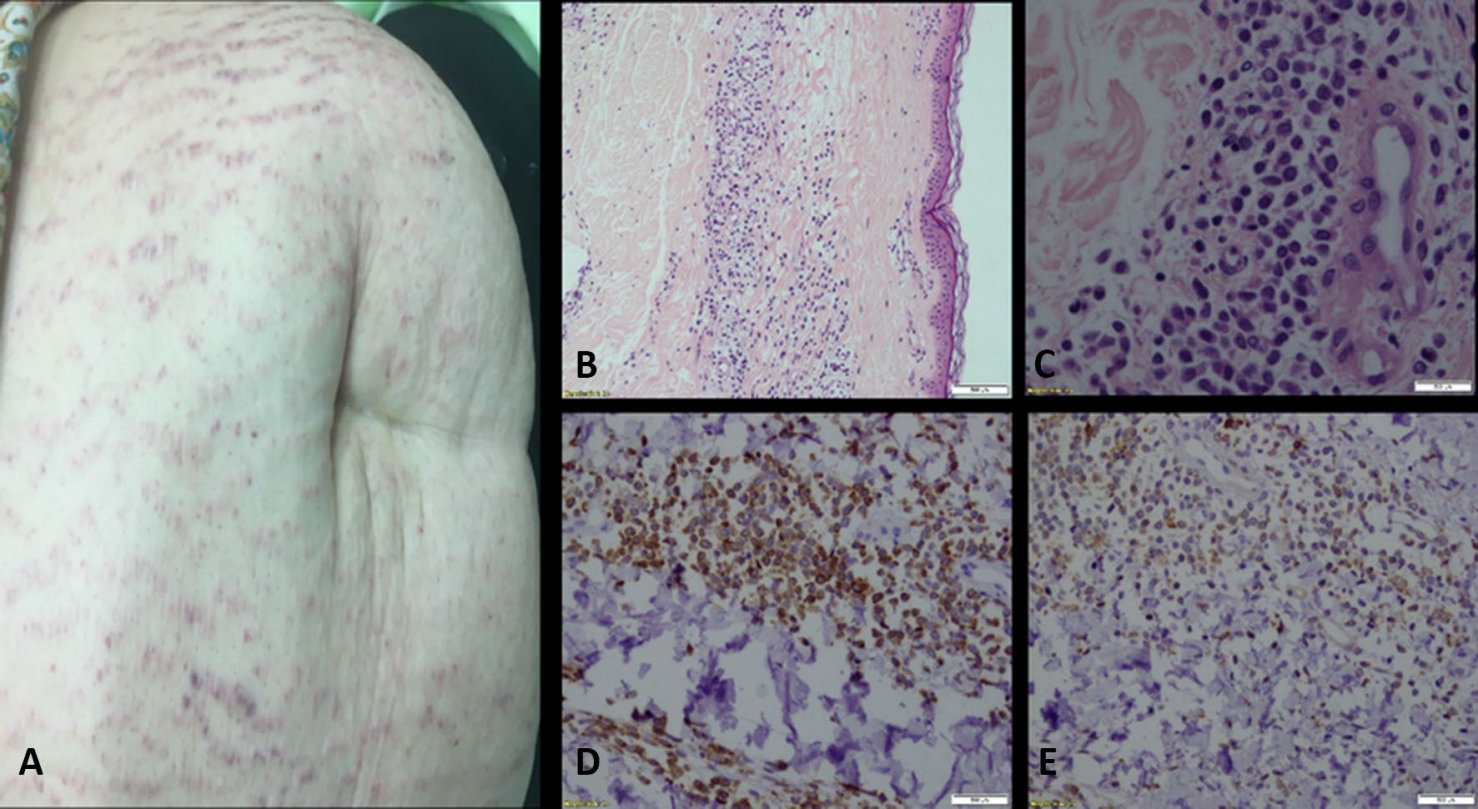
Leukemia cutis. A 42-year-old woman with AML who underwent biopsy of a skin eruption. (**A**) The patient presented with hyperleukocytosis and abdominal purple striae-like infiltrated plaques in a reticulated pattern. (**B**,**C**) A punch biopsy performed from the right abdomen revealed a mild perivascular infiltrate in the dermis, composed of medium-sized immature atypical cells, otherwise unremarkable. The epidermis shows no significant changes; note the subepidermal Grenz zone (H&E, X100). (**C**) Same as (**B**) at higher magnification (H&E X400). (**D**,**E**) The atypical cells stain positive for CD45 (X200) (**D**) ,CD4 (X200)) **E**) ,HLA-DR, CD11c, CD68 and lysozyme. Cells are negative for CD3, CD20, CD79a, CD34, CD138, TdT, CD30, CD123 and cytokeratin. Ki67 shows a proliferative index of 25% in the infiltrates. The overall histological findings together with the immunohistochemical stains are compatible with AML involving the skin (leukemia cutis).

A representative case of one of the patients in our cohort is presented in Fig. 1. The patient was a 42-year-old woman who presented to the hospital with 3 weeks of fever, sore throat, weakness, weight loss and new abdominal purple striae-like infiltrated plaques in a reticulated pattern. Blood tests revealed hyperleukocytosis (defined as extreme leukocytosis), thrombocytopenia of 81,000/microL and multiple myeloblasts on peripheral blood smear and bone marrow aspirate. A 4-mm punch biopsy of the abdominal stria demonstrated mild to moderate perivascular infiltrates of immature medium sized atypical cells (CD45+) in the dermis, compatible with leukemia cutis. This was the only patient in whom skin biopsy caused complications, consisting of infected hemorrhagic bulla at the biopsy site and systemic fever. Treatment consisted of the administration of broader empirical antibiotic treatment with vancomycin in order to cover resistant nosocomial gram-positive pathogens residing in the skin. In this case, the skin biopsy result of leukemia cutis did not directly alter the patient’s management since the diagnosis of AML was already known. However, biopsy of this atypical skin eruption enabled exclusion of other etiologies, such as infections or Sweet syndrome, which would alter patient’s immediate care.

## Discussion

We found that skin punch biopsy was a relatively common and safe procedure in patients with AML under intensive chemotherapy. Although biopsies infrequently affected immediate management, they did provide potentially valuable information for long term patient care and enabled exclusion of differential diagnoses. A total of 37 skin biopsies were performed in 35 of 216 patients with AML admitted to our institute during the 10-year study period (18%). The skin eruptions were frequently mild and usually appeared after commencement of chemotherapy. Drug eruption was the most prevalent etiology identified. In most patients (92%), histological and tissue culture results did not directly alter patient management. Of note, in all 3 patients for whom skin biopsy results did change immediate management, the procedure was performed during AML chemotherapy and not prior to therapy. Nonetheless, information obtained from 8 additional biopsies (21.6%), with leukemia cutis and non-contaminant skin cultures, could have potential effect on long term management. In the case of leukemia cutis, although the immediate induction chemotherapy would probably remain unchanged, the diagnosis of extramedullary leukemia in the skin could have an effect on future decisions regarding consolidation chemotherapy and/or transplantation.

For infectious rash etiologies, even if immediate treatment choice is not affected, this could lengthen the necessary duration of antibiotic treatment, and perhaps affect future choice of antibiotics.

The skin eruptions were associated with fever in almost half the patients, and most of the biopsies were performed at the time the patients had severe neutropenia and moderate-to-severe thrombocytopenia. Nonetheless, in the vast majority of cases, the skin biopsies were uneventful, and no excessive risk of complications was evident; only one patient had mild bleeding at the biopsy site with local infection. The low rate of bleeding complications among the thrombocytopenic patients with AML in our cohort is similar to the low rate of hemorrhagic complications (0.49%) reported by Xia et al.^13^ following diagnostic skin biopsy in a cohort of thrombocytopenic inpatients.

Currently, there is no platelet transfusion threshold for performing a skin biopsy, and we are not aware of previous reports in the literature regarding the safety of this procedure in the setting of intensive chemotherapy for AML. However, there are reports of the safety of other procedures in patients with acute leukemia. Lumbar puncture, for example, has been associated with a low rate of bleeding complications in both pediatric^14^ and adult^15^ patients, with a platelet threshold of 10,000/microL and 20,000/microL, respectively. Nonetheless, the American Association of Blood Banks suggests prophylactic platelet transfusion for patients undergoing elective diagnostic lumbar puncture when the platelet count is less than 50,000/microL^16^.

The importance of a skin biopsy in AML patients stems from the necessity to exclude certain diagnostic possibilities in these selected patients, which may alter the course of treatment. The differential diagnosis of skin eruption in treated patients with AML is diverse, partially due to the immunosuppressed state induced by the hemato-oncological disease as well as the drugs used. Possible etiologies include extramedullary infiltration of the primary disease (leukemia cutis), paraneoplastic syndrome, drug eruption (due to chemotherapy, antibiotics etc.), infection (local skin infection, dissemination of a bacterial/fungal/viral source of infection) or an inflammatory disorder (i.e. Sweet syndrome, acral erythema of chemotherapy)^3–8^. The majority of data in the literature regarding skin eruptions in AML consists of case reports of leukemia cutis and drug eruptions in the context of high-dose chemotherapy. Cytarabine, a main constituent of both induction and consolidation regimens for AML, is notoriously associated with different types of skin eruptions^3–8^. In accordance with the literature, in our study, drug reaction, involvement by the primary hematological disease and infection were the leading findings on histopathological study and tissue cultures of the skin biopsies.

Wright et al.^9^ reported a high prevalence of skin eruptions amongst hemato-oncological patients (14/127, 11%). The highest rate was found in patients with newly diagnosed AML who received induction protocols containing cytarabine (55%). Pearson et al.^10^ reported a 38% incidence of rashes amongst 84 hemato-oncological patients during 200 hospitalization episodes. Acral erythema was the most frequent rash observed. The authors stated that skin biopsies were rarely conducted in the study population, although the exact number and the biopsy findings were not mentioned.

The main limitation of our study is the single-center retrospective design with a limited number of patients and skin biopsies. Therefore, generalization of results is limited. In addition, in daily practice, biopsy results are often delayed, necessitating management decisions as well as empirical treatment according to clinical judgment. Nonetheless, to the best of our knowledge, this is the first study aiming to clarify the clinical uncertainty of the yield and safety of biopsies from skin eruptions in patients with AML during hospitalization for intensive chemotherapy treatment.

To conclude, with the limitation of a retrospective analysis, this study shows that skin biopsies from patients with AML undergoing in-hospital intensive chemotherapy is relatively safe. Although immediate patient management is usually not affected, biopsies provide valuable information for long term management in a substantial portion of patients. Thus, unless rash etiology is clearly evident, our results suggest that skin biopsies have considerable diagnostic yield.

## Data Availability

The datasets generated during and/or analysed during the current study are available from the corresponding author on reasonable request.

## Abbreviations

AML: Acute myeloid leukemia
CTCAE: Common Terminology Criteria for Adverse Events
